# How to target small cell lung cancer

**DOI:** 10.18632/oncoscience.212

**Published:** 2015-08-21

**Authors:** Gerhard Hamilton, Barbara Rath, Ernst Ulsperger

**Affiliations:** ^1^ Ludwig Boltzmann Cluster of Translational Oncology, A-1090 Vienna, Austria

**Keywords:** small cell lung cancer (SCLC), circulating tumor cells (CTCs), secretome, chitinase 3-like1 (CHI3L1), YKL-40, chronic obstructive pulmonary disease (COPD), prognostic marker, Inflammation

## Abstract

Small cell lung cancer (SCLC) is a highly malignant disease with dismal prognosis. Although great progress has been made in investigating genetic aberrations and putative drivers of this tumor entity, the mechanisms of rapid dissemination and acquisition of drug resistance are not clear. The majority of SCLC cases are characterized by inactivation of the tumor suppressors p53 and retinoblastoma (Rb) and, therefore, interchangeable drivers will be difficult to target successfully. Access to pure cultures of SCLC circulating tumor cells (CTCs) and study of their tumor biology has revealed a number of new potential targets. Most important, expression of chitinase-3-like-1/YKL-40 (CHI3L1) which controls expression of vascular epithelial growth factor (VEGF) and matrix metalloproteinase-9 (MMP9) was newly described in these cells. The process switching CHI3L1-negative SCLC cells to CHI3L1-positive CTCs seems to be associated with cytokines released by inflammatory immune cells. Furthermore, these CTCs were found to promote monocyte-macrophage differentiation, most likely of the M2 tumor-promoting type, recently described to express PD-1 immune checkpoint antigen in SCLC. In conclusion, dissemination of SCLC seems to be linked to conversion of regular tumor cells to highly invasive CHI3L1-positive CTCs, which are protected by immune system suppression. Besides the classical targets VEGF, MMP-9 and PD-1, CHI3L1 constitutes a new possibly drugable molecule to retard down dissemination of SCLC cells, which may be similarly relevant for glioblastoma and other tumor entities.

## INTRODUCTION

Lung cancer is the leading cause of cancer-related deaths worldwide and, in particular, small cell lung cancer (SCLC) constitutes a highly aggressive variant accounting for approximately 15% of cases [1 - 3]. This tumor entity is characterized by early dissemination and, therefore, the majority of patients is not eligible for surgical treatment and is treated by systemic chemotherapy [4]. Despite excellent initial response rates to platinum-based combination chemotherapy patients relapse within approximately one year and are not amenable to further effective therapy [5, 6]. A host of chemotherapeutics and targeted drugs have shown no advantage over standard therapy [7]. In contrast to other tumor entities, SCLC has been reported to exhibit high numbers of circulating tumor cells (CTCs) in blood [8, 9]. CTCs are shed from primary tumors or metastatic sites and may be used as so-called liquid biopsies for early diagnosis, risk evaluation and monitoring of therapy [10, 11]. In SCLC patients, the detection rates of the CellSearch^TM^ system which counts EpCAM-positive tumor cells ranged from 14 to 70% [8]. A minority of these disseminated cells can develop into metastases and most of the cells persist in blood only for a short time and undergo apoptosis [10].

Molecular characterization of CTCs would require expansion of CTCs in cell culture systems, so far only achieved for a few breast cancer CTCs and one colon-derived line [12, 13]. We collected peripheral blood of SCLC patients at different stages, enriched CTCs by Ficoll–Hypaque density gradient centrifugation and obtained several SCLC CTC cell lines upon culture in serumfree RPMI-1640 medium supplemented with insulin, IGF-1, transferrin and selenite [14]. Proliferating CD56-positive CTC cultures from relapsed SCLC patients displayed typical loosely attached or compact spheroid morphology. Access to expanded SCLC CTCs allowed for a detailed study of the cell biology of this invasive population for the first time.

### Expression of chitinase-3-like-1 by SCLC CTCs

Screening of the permanent CTC cultures of patients for secreted cytokines revealed presence of chitinase 3-like1 (CHI3L1)/YKL-40, known to be upregulated in a range of tumor entities and to be associated with increased metastasis and decreased survival [15, 16]. The alternative designation of CHI3L1, namely YKL-40, is based on the first three N-terminal amino acids, tyrosine (Y), lysine (K) and leucine (L) and the apparent molecular weight of YKL-40 and is here not used furthermore [17]. This protein lacks enzymatic activity and its mechanism of promoting tumor dissemination has not been resolved. Results from SCLC CTC cultures suggest CHI3L1 as marker and important effector of tumor cell dissemination in the peripheral blood. Furthermore, this protein may link chronic inflammation of the lung, chronic obstructive pulmonary disease (COPD) and lung cancer.

Levels of circulating CHI3L1 are increased in many malignancies, including cancers involving the lung, prostate, colon, rectum, ovary, kidney, breast, glioblastomas, and malignant melanoma [17]. Numerous studies have correlated elevated serum levels of CHI3L1 with poor prognosis and low survival in patients suffering from these malignancies. Furthermore, in breast and colon cancer it was shown that increased CHI3L1 levels correlate with tumor grade and poor differentiation of cancer cells [18, 19]. CHI3L1 was found to promote cancer cell proliferation, macrophage recruitment and angiogenesis [20]. CHI3L1 protein overexpression and high microvascular density (MVD) were significantly associated with tumor relapse and an unfavorable overall survival in NSCLC, hepatocellular carcinoma, glioblastoma, cervical and renal cancer [21 - 25]. In summary, the CHI3L1 protein is expressed in many types of cancer cells and its highest plasma levels have been found in patients with metastatic disease, short recurrence/progression-free intervals, and low overall survival [16, 26, 27]. Furthermore, serum CHI3L1 was evaluated in 131 patients with SCLC and increased levels were associated with increased hazard for death [28]. Elevated serum levels of CHI3L1 have been correlated with poor prognosis and shorter survival of patients with cancer and inflammatory diseases. The biological and physiological functions of CHI3L1 in cancer causing dissemination of tumor cells have not yet been elucidated [17, 29].

### Role of mammalian chitinases

Chitinase enzymes hydrolyse the polysaccharide chitin, an abundant architectural component in invertebrates and fungi. Most mammals encode at least two endochitinases (CHIT1 and CHIA/AMCase), as well as several homologues encoding catalytically inactive chitinase-like proteins in numerous pathological conditions [30]. Expansion and selection of chilectins is found in mammals and a function in immunity proposed for CHI3L1. Mammals are not known to synthesize chitin or metabolize it as a nutrient, yet the human genome encodes eight glycoside hydrolase family 18 chitinases (GH18) family members, obviously fulfilling alternative functions [31, 17]. A process of gene duplication and diversification has resulted in multiple functions that are not related to nutrient utilization or growth-related turnover of chitinous structures. GH18 proteins which lack an essential catalytic glutamic acid that donates a proton required for hydrolytic enzyme activity are likely to act as lectins. Three human genes (OVGP1, CHI3L1 and CHI3L2) encode chitolectins likely to be involved in tissue remodeling during inflammation and/or development. A number of cell types, including macrophages, dendritic cells, chondrocytes and synovial cells secrete CHI3L1 and CHI3L2 proteins under inflammatory conditions [17]. Association of GH18 chitinase activity with Th2-type cell inflammation suggests a role at the interface of innate and adaptive immunity.

CHI3L1 interacts with glycosaminoglycans, such as heparin and hyaluronan, and binds to collagen type I, II, and III [17]. CHI3L1 is a secreted 40 kDa glycoprotein which lacks chitinase activity due to mutations within its active site [32]. CHI3L1 has been linked to activation of the AKT pro-survival (anti-apoptotic) signaling pathway and enhances tumor survival in response to irradiation which is frequently applied prophylactically in SCLC [33]. CHI3L1 suppression by shRNA reduced glioma cell invasion and anchorage-independent growth and increased cell death in response to several anticancer drugs, including cisplatin, etoposide and doxorubicin [23]. The CHI3L1 protein was significantly elevated in human serum samples from all types of lung cancers and recombinant CHI3L1 stimulated proliferation and growth of Lewis lung carcinoma cells [34, 35].

CHI3L1 is detectable in a variety of normal cells, including macrophages, neutrophils, epithelial cells, smooth muscle cells, and chondrocytes, and its expression is stimulated by a number of cytokines, including IL13, IL6, IL1β, and IFNγ [36]. Elevated serum levels of CHI3L1 have been associated with a negative outcome in a number of nonmalignant diseases such as inflammation and asthma [17]. Additionally, CHI3L1 may link inflammation of bronchial tissue, COPD and lung cancer and provide an explanation for COPD as risk factor of lung cancer [37]. The presence of CTCs as examined by an ISET filtration-enrichment technique (Isolation by Size of Tumor cells) in COPD patients (3% positivity) was reported to precede the clinical detection of early stage lung cancer by 1 – 4 years [38]. CHI3L1-positive tumor cells, exhibiting altered regulation of VEGF and MMP9, decreased expression of cadherin and increased cell motility, display the combination of characteristics required to generate metastases as CTCs [17]. Our results demonstrating CHI3L1 as part of the secretome of *ex vivo* expanded SCLC CTCs point to these disseminated tumor cells as partial source of this protein directly produced in blood [15].

### CHI3L1 and inflammation in bening diseases

COPD is a complex inflammatory disease involving several types of inflammatory cells and multiple inflammatory mediators. Abnormal numbers of inflammatory cells such as macrophages, dendritic cells, neutrophils, and T lymphocytes have been documented in COPD [39]. Acidic mammalian chitinase (AMCase) inhibits chitin-induced innate inflammation, allergen-induced Th2 inflammation and mediates effector functions of IL-13. AMCase induces airway hyperresponsiveness in allergic asthma patients. CHI3L1 inhibits oxidant-induced lung injury, augments adaptive Th2 immunity, regulates apoptosis, stimulates alternative macrophage activation and contributes to fibrosis and wound healing [40]. CHI3L1(−/−) animals have markedly diminished antigen-induced Th2 responses and epithelial CHI3L1 rescues the Th2 responses in these animals [41]. The ability of IL13 to induce tissue inflammation and fibrosis was also markedly diminished in the absence of CHI3L1. This protein also inhibit inflammatory cell apoptosis/cell death while inhibiting Fas expression. Significantly higher serum levels of CHI3L1 in samples from smokers and COPD patients compared to non-smokers indicate an important role in pulmonary inflammation and emphysematous alterations [42]. The related CHIT1-1 is expressed by phagocytic cells [43]. Furthermore, CHI3L1 protein is expressed in colonic epithelial cells and macrophages in the inflamed colon of colitis [20].

COPD and lung cancer are closely related [44]. The annual incidence of lung cancer arising from COPD has been reported to be 0.8-1.7 %. Inflammatory mediators may promote the growth of bronchioalveolar stem cells in the development of lung cancer from COPD. The close association between the two diseases is independent of the smoking history [45].

### CHI3L1 and inflammation in malignant diseases

Elevated serum CHI3L1 levels in patients with metastatic cancers, including SCLC, are associated with poor prognosis, although SCLC cell lines display no or very limited CHI3L1 expression [46]. In most biopsies from SCLC patients, CHI3L1 mRNA transcripts were positive but the signal was localized in macrophages in the peritumoral stroma. No CHI3L1 mRNA expression was found in the cancer cells, in macrophages infiltrating the solid tumor areas, or in non-malignant tissue. Inflammation is a hallmark of cancer put into execution by immune cells which exert either pro- or antitumor properties and alter resistance to therapy [47]. Macrophages originate from monocytic precursors in the blood and undergo specific differentiation in tumors to the so-called suppressor M2 type, which has poor antigen-presenting capacity, impairs T-cell activation and fuels angiogenesis and metastasis [48]. Tumor-derived cytokines such as IL-4, IL-10, IL-13, TGF-β, or prostaglandin E2 promote M2 generation and are correlated to poor prognosis in several human cancers [49 - 51]. Metastasis is initiated by digestion of extracellular matrix with matrix metalloproteinases (MMPs) and stimulation of angiogenesis via VEGF production [52, 53]. In detail, tumor-associated macrophages (TAMs) isolated in NSCLC and kept in short-term culture expressed high levels of Cathepsin K, COX-2, MMP-9, PDGF-B, uPA, VEGF, and HGF [54]. The increase of invasiveness in the lung cancer cell lines was also correlated with their MMP9 activity. The M2-type TAMs in lung cancer release IL-10 and TGFβ [55, 56]. IL-10-positive xenografts showed significantly increased tumor growth fractions and increased angiogenesis. Expression of the cognate receptor, IL-10R, was detected in approximately 20% of NSCLC xenografts and IL-10R mRNA expression in almost all cases of NSCLC [57]. The NSCLC patients with IL-10 production showed a significantly poorer prognosis [58].

Tumor cell dissemination to distant organ sites is a complex process involving multiple cell types, soluble growth factors, adhesion receptors, and tissue remodeling [59]. In tumor biology, it has been increasingly appreciated that MMP-9 from inflammatory cells, particularly neutrophils, codetermines prognosis and outcome [60]. MMP-9 is induced by many pro-inflammatory cytokines. MMP9-expressing tumor-associated macrophages play a key role in preparing premetastatic sites for eventual malignant cell growth in a manner dependent upon vascular endothelial growth factor receptor-1 (VEGFR-1) [61]. Whereas TAMs may kill neoplastic cells following activation by IL-2, IFN, or IL-12, they produce a number of potent angiogenic and lymphangiogenic growth factors, cytokines, and proteases that potentiate neoplastic development. Angiogenesis, i.e. the formation of new blood vessels, is important for lung tumor growth, invasion and metastasis [62]. The most studied anti-angiogenic agents include anti-VEGF monoclonal antibodies and VEGF receptor tyrosine kinase inhibitors [63]. CHI3L1 induces angiogenesis in vitro and in animal tumor models and is expected to promote evasion of CTCs from primary sites [64]. A CHI3L1-neutralizing monoclonal antibody blocks tumor angiogenesis and progression. When CHI3L1 expression of a glioblastoma cell line was inhibited, VEGF production was reduced but blockade of VEGF induced the expression of CHI3L1 [17]. Thus, inhibition of VEGF by antibodies or small molecule inhibitors may be counterproductive in SCLC.

### Induction of expression of CHI3L1 in SCLC CTCs

The proinflammatory cytokines, such as IL-1, TNF-α, and IL-6, modulate CHI3L1 expression in chondrocytes, macrophages, and also glioblastoma cells [65, 66]. In chondrocytes, the inflammatory cytokines TNF-α and IL1 potently induced steady-state levels of CHI3L1 mRNA and protein secretion. Cytokines of the IL-6 family activate STAT3 *in vitro* [67]. Nuclear factor I-X3 (NFI-X3) and STAT3 form a complex that binds to regulatory elements in the CHI3L1 promoter and activates transcription [68]. In contrast to other cancer types, TNFα suppresses CHI3L1 expression in glioma cell lines in a NF-κB-dependent manner. Even though TNF-alpha causes recruitment of p65 and p50 subunits of NF-κB to the CHI3L1 promoter in all cell types, recruitment of histone deacetylases (HDAC)-1 and -2, and a consequent deacetylation of histone H3 at the CHI3L1 promoter occurs only in glioma cells [66]. The IL6 family of cytokines share gp130 as a signal-transducing receptor component and oncostatin M, a member of this family, exerts STAT3 activation [67]. The IL-1-induced RelB/p50 complex formation was further promoted by oncostatin M and that these complexes directly bound to the CHI3L1 promoter [69]. Expression of RelB was strongly upregulated during inflammation *in vivo* and by IL-1 in astrocytes *in vitro*. Thus, IL-1 and the IL-6 family of cytokines regulate CHI3L1 expression during inflammation via both STAT3 and RelB/p50 complexes.

**Figure 1:**
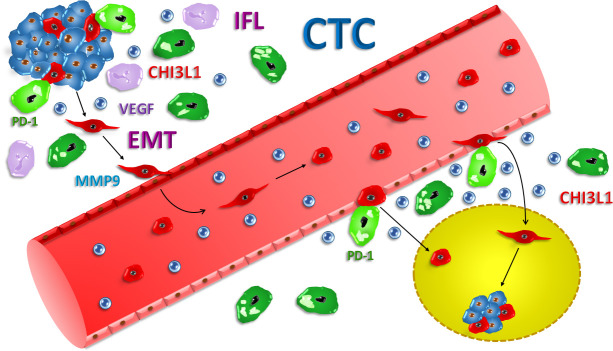
Proposed model of SCLC CTC dissemination SCLC is initiated by a series of mutations concerning p53 and Rb tumor suppressors as well as one of several oncogenes which function as tumor drivers. Factors released by the tumor cells attract immune effector cells causing local inflammation (IFL). Tumor-associated macrophages (TAM) secrete cytokines which promote cancer cell growth and seem to induce CHI3L1 in putative precursors of CTCs. Furthermore, TAMs express immune checkpoint proteins (light green macrophages), such as PD-1/PD-L1, which provide suppression of antitumor lymphocyte responses. The few susceptible SCLC cells (red) which acquired the ability to produce CHI3L1 and express VEGF and MMP9 enter the bloodstream, possibly after epithelial-to-mesenchymal transition (EMT). CTCs reach secondary sites for invasion and formation of metastases. Their production of CHI3L1 leads to monocyte-macrophage differentiation and these new suppressive TAMs may shield the disseminated SCLC cells against attacks of the immune system, such promoting formation of secondary tumor lesions.

pSTAT3 overexpression is an important factor related to prognosis of NSCLC patients [70]. Similarly, the expression levels of STAT3, pSTAT3, and VEGF-C were higher in 128 cases of SCLC than in 40 normal tissues [71]. These markers showed positive correlations with lymph node metastasis, clinical stage, tumor size and overall survival rates. Another group reported that STAT3 is constitutively phosphorylated in SCLC and is important in SCLC growth and spreading [72]. Thus, induction of expression of CHI3L1 in SCLC CTCs stems most likely from exposure of a fraction of the tumor cells to inflammatory cytokines produced by macrophages in the adjacent stroma tissue. STAT3 constitutes a putative important mediator of this process. CHI3L1-positive tumor cells armed with MMP9 and VEGF seem capable to pass the neighboring matrix and enter the bloodstream, possibly via newly formed microvessels (Figure 1). This mechanism of tumor escape and dissemination may not be limited to SCLC, since similar effectors and processes are reported for other tumor entities like the highly invasive glioblastoma.

### SCLC and immunotherapy

The immune checkpoint proteins, including the B7/CD28 receptor superfamily, have become increasingly important targets for pharmacologic blockade. Several classes of new agents have impressive clinical activity in melanoma and lung cancer [73, 74]. None of the SCLCs showed PD-L1 protein expression in tumor cells [75]. PD-L1 and PD-1 expression was noticed in the stroma: approximately 20% of cases showed PD-L1 expression in tumour-infiltrating macrophages and approximately 50% showed PD-1 positive tumor-infiltrating lymphocytes. Thus, the PD-1/PD-L1 pathway seems to be activated in a fraction of SCLCs. Gliomas, which express CHI3L1, are known to induce local and systemic immunosuppression, inhibiting T-cell-mediated cytotoxic responses to tumor growth. In contrast to many other monocyte/macrophage markers, CHI3L1 expression is absent in monocytes and strongly induced during late stages of human macrophage differentiation [76]. Gliomas can upregulate B7-H1 expression in circulating monocytes and tumor-infiltrative macrophages through modulation of autocrine/paracrine IL-10 signaling, resulting in an immunosuppressive phenotype [77]. We have found that cocultures of SCLC CTCs with normal white blood cells lead to monocyte-macrophage differentiation (manuscript in preparation). Such recruitment of macrophages by the CHI3L1-positive CTCs may induce M2-type local immunosuppression and protect the disseminated SCLC tumor cells from attack by the immune system [78].

### Targeting of CHI3L1

CHI3L1 suppressed E-cadherin but induced MMP9 and cell motility in glioma cells, all of them essential features of tumor cell invasion [79]. Targeting CHI3L1 with neutralizing antibodies has been proven effective as treatment of gliomas in animal models [80]. In good agreement, CHI3L1 suppression by shRNA reduced glioma cell invasion, anchorage-independent growth and increased cell death in response to several anticancer drugs, including cisplatin, etoposide and doxorubicin [23]. Overactivation of the STAT3 pathway in lung alveolar type II epithelial cells was described to induce chronic inflammation and adenocarcinoma in the lung of bitransgenic mice and downstream-regulated CHI3L1 showed increased concentration in bronchioalveolar lavage fluid [81]. Thus, effects of the STAT3 inhibitor STX-0119 on effects of CHI3L1 were studied [82]. Whereas this inhibitor lacked significant effects on growth of a temozolomide-resistant U87 glioblastoma cell line, it suppressed the growth of this tumor in nude mice by more than 50%, and prolonged survival. Accordingly, significantly decreased CH3L1 levels were found in cell supernatants of the resistant U87 cell line in response to STX-0119.

CHI3L1 has retained its chitosan binding activity. Correspondingly, reports indicate that N-acetyl-d-glucosamine oligomers (chitin oligosaccharide; NACOS) and d-glucosamine oligomers (chitosan oligosaccharide; COS) have activities against cancer and inflammation [83, 84]. COS significantly inhibited cell proliferation of the hepatocellular carcinoma cell line HepG2 and intraperitoneal injections of COS delayed the growth of HepG2 xenografts in immunodeficient mice [85]. Moreover, MMP9, an enzyme associated with metastasis, could be inhibited by COS in Lewis lung carcinoma and in corresponding xenografts. COS administration inhibited tumor growth, decreased the number of metastatic colonies in lung, and prolonged survival time. In another model, decreased tumor dissemination could also be demonstrated in response to administration of chitosan nanoparticles [86].

Employing structural chitinase data, the inhibitor bisdionin C which shows activity in the submicromolar range against GH18 enzymes was designed [87]. A crystallographic structure of a chitinase-bisdionin C complex demonstrated that two aromatic systems of the ligand block two conserved tryptophan residues of the active site chitinases. The AMCase inhibitor bisdionin F alleviated allergic inflammation in experimental animals; however dramatic neutrophilia in the lungs was observed as side effect [88]. Therefore, the current bisdionin drugs are not expected to work in clinics but may serve as lead compounds for development of chitinase inhibitors for the clinics.

## DISCUSSION

In contrast to non-small lung cancer (NSCLC), targeted therapy directed to specific oncogenes is not available for SCLC and platinum-based combination chemotherapy with etoposide and second-line topotecan are standard care [5, 6]. SCLCs invariably show inactivation of p53 and retinoblastoma (Rb) and in the absence of these two tumor suppressors several alternative growth factor pathways induce vigorous progression [89]. Despite an initial high response rate, tumors relapse early and are not amenable to effective further therapy. In most cases CTCs are scarce and investigations are restricted to single-cell genetic analysis and detection of surface markers or a few cytokines [11] Ubiquitous expression of CHI3L1 in a range of solid tumors, as indicator of early dissemination and lower overall survival, seems to be partially due to CHI3L1-positive CTCs providing an explanation for the correlation of this marker with tumor dissemination [15, 17]. Furthermore, several findings indicate an association of CHI3L1 expression with chemoreistance. It should be investigated whether CHI3L1 constitutes a general CTC-associated marker, aside from SCLC, and whether CTC counts parallel the serum concentrations of this antigen. Expression of CHI3L1 by SCLC CTCs is suggested to be acquired in peritumoral stroma in response to inflammatory cytokines produced by TAMs. CHI3L1 controls MMP9 and VEGF and induces an invasive phenotype that may be targeted by inhibitors aiming at induction, expression and/or function of this secreted factor. The pathologist Rudolf Virchow stressed the importance of inflammatory processes for cancer in 1863, although they are not involved in initiation of malignant diseases, as he thought, but in maintenance of the tumors and most likely in arming and shielding of CTCs [90, 91]. As SCLC is just an example of a highly invasive cancer the proposed model may hold true for other tumor entities, especially in the light of several similar features observed in invasive glioblastoma such as CHI3L1-positivity and recruitment of myloid-derived suppressor cells [92, 93].
